# A systematic review of the mechanism of action and potential medicinal value of codonopsis pilosula in diseases

**DOI:** 10.3389/fphar.2024.1415147

**Published:** 2024-05-13

**Authors:** Huina Guo, YiChen Lou, Xiaofang Hou, Qi Han, Yujia Guo, Zhongxun Li, Xiaoya Guan, Hongliang Liu, Chunming Zhang

**Affiliations:** ^1^ Shanxi Key Laboratory of Otorhinolaryngology Head and Neck Cancer, First Hospital of Shanxi Medical University, Taiyuan, China; ^2^ Shanxi Province Clinical Medical Research Center for Precision Medicine of Head and Neck Cancer, First Hospital of Shanxi Medical University, Taiyuan, China; ^3^ The First Clinical Medical College of Shanxi Medical University, Taiyuan, China; ^4^ Department of Otolaryngology Head and Neck Surgery, First Hospital of Shanxi Medical University, Taiyuan, China; ^5^ Department of Cell Biology and Genetics, The Basic Medical School of Shanxi Medical University, Taiyuan, Shanxi, China

**Keywords:** codonopsis pilosula (CP), combination drugs, active ingredients, diseases, cytoscape

## Abstract

As a traditional Chinese medicinal herb with a long history, Codonopsis pilosula (CP) has attracted much attention from the medical community in recent years. This review summarizes the research progress of CP in the medical field in the past 5 years. By searching and analyzing the literature, and combining with Cytoscape software, we comprehensively examined the role and mechanism of action of CP in individual application, combination drug application, and the role and mechanism of action of codonopsis pilosula’s active ingredients in a variety of diseases. It also analyzes the medicinal use of CP and its application value in medicine. This review found that CP mainly manifests important roles in several diseases, such as cardiovascular system, nervous system, digestive system, immune system, etc., and regulates the development of many diseases mainly through the mechanisms of inflammation regulation, oxidative stress, immunomodulation and apoptosis. Its rich pharmacological activities and diverse medicinal effects endow CP with broad prospects and application values. This review provides valuable reference and guidance for the further development of CP in traditional Chinese medicine.

## 1 Introduction

Codonopsis pilosula (CP), a plant belonging to the family Codonaceae of the order Platycodonopsis, has more than 60 species. These perennial plants are mainly found in East, Southeast and Central Asia (Bailly, 2021). The term ginseng is named after the plant’s shape and medicinal value. “Dang” in Chinese pharmacology refers to a medicine with a tonic. “Shen” refers to the shape of the plant’s rhizome, which resembles a human body, while “Dang Shen” in Chinese pharmacology relates to a drug with a tonic effect. Therefore, the name “Dangshen” is intended to convey the combination of the morphology of the root of CP and its tonic effect. Since the Qing Dynasty (Gao et al., 2018), CP has been used as a traditional Chinese medicine for thousands of years, and it is widely used in medicine in China, Japan, Korea and other countries (Luan et al., 2021).

As a valuable botanical herb, CP is highly regarded for its unique medicinal value and health effects (Dong et al., 2023). Sweet in flavor and neutral in nature, CP returns to the spleen and lung meridians (Lan et al., 2023), and can be applied alone (Wang J. et al., 2024) or used in combination with other medicines (Zhao et al., 2024), its main effects include invigorating the spleen and benefiting the lungs, invigorating blood circulation and removing blood stasis. It has significant roles in immunity, hematopoiesis, gastrointestinal and endocrine aspects. However, with the continuous development of modern pharmacology, studies have gradually revealed the critical role of CP in the fields of neuroprotection, anti-aging, antioxidant and antitumor (Lan et al., 2023). CP is rich in polysaccharides, ginsenosides, alkaloids, flavonoids and other complex active ingredients (Gao et al., 2018; Bailly, 2021; Luan et al., 2021). These active ingredients have a wide range of roles in the digestive system, metabolic system, nervous system, cardiovascular system and cancer treatment. For example, Codonopsis polysaccharides (CPPs), found in CP, is thought to play a role in increasing splenic tone (Cao et al., 2022). However, the mechanism of action of CP in disease treatment, whether used alone or in combination with other drugs, is unclear.

**FIGURE 1:**
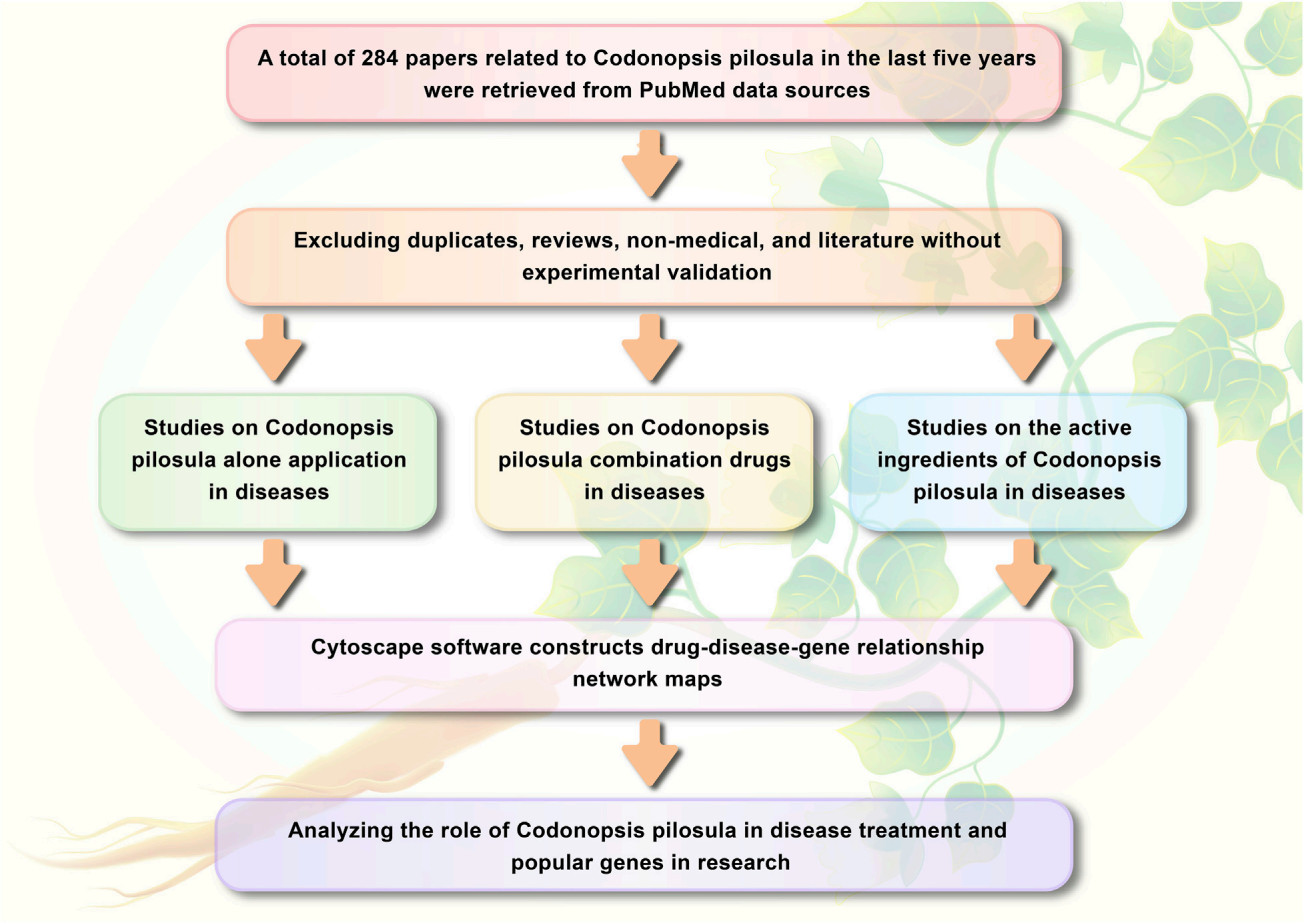
Article statey and analysis flowchart.

In this review, a total of 284 literature related to CP in the last 5 years were obtained by searching through PubMed data sources. After excluding duplicates, reviews, non-medical, and studies without experimental validation, PubMed, CNKI, GeenMedical, and Ablesci were utilized to assist in obtaining the full text of the articles. After reading the articles to obtain key information, the literature was categorized into three groups: 20 studies on CP alone application in diseases, 32 studies on CP combination drugs in diseases, and 52 studies on CP active ingredients in diseases. The cytoscape software was used to construct drug-disease-target relationship network diagrams and analyze their topology, so as to analyze the extensiveness of CP in the treatment of diseases and the popular genes in research, with a view to providing a more in-depth understanding of CP in medical research. The research idea is shown in Figure 1.

## 2 Subsections relevant for the subject

### 2.1 Studies on CP alone application in diseases

Chinese medicinal herb CP is certified by the National Geographical Indication of China as a valuable medicinal herb. It has the effects of strengthening the spleen, moistening the lungs, activating the blood, and generating fluids (Wang J. et al., 2024). It is often studied by researchers as a stand-alone agent in addition to being used in combination with other drugs (Cao et al., 2022). We summarize the research progress of CP in diseases in the last 5 years. The results (Table 1) showed that CP is mainly used in the treatment of diseases of the nervous system, digestive system, and immune system. Chronic cerebral ischemia is a symptom of brain supplemental hypoxia caused by long-term blood supply insufficiency, and CP can improve cerebral blood circulation and reduce ischemic damage, which alleviates chronic cerebral ischemia (Wang J. et al., 2024). Alzheimer’s disease (AD) is a gradual progressive neurological disorder, and CP can be used to improve nerve cell function and reduce neurodegeneration, which helps to slow down the development of Alzheimer’s disease (Xie et al., 2024). Colon cancer (Cao Y. et al., 2019), gastric precancerous lesions (He et al., 2022), liver cancer (Liu Z. et al., 2022; Li N. et al., 2024), hepatitis (Zeng et al., 2023), and colitis (Li F. et al., 2024) are malignant tumors or inflammatory diseases of the digestive system, and CP plays an important role in preventing and treating these diseases play an important role. Interestingly, CP can also regulate the immune function of sepsis (Choi et al., 2023), rheumatoid arthritis (Wang Y.-J. et al., 2022), allergic asthma (Seo et al., 2019), and other immune system disorders (Zou et al., 2019), reduce inflammatory reactions, and control the development and symptoms of diseases.

**TABLE 1:** Studies on CP alone application in diseases.

References	Related targets and genes	Functioning diseases
Wang et al. (2024a)	CKLF1, HIF-1α	Chronic cerebral ischemia
Li et al. (2024a)	GSH, MPO, SOD, MDA, AKT, p-PI3K, Bcl2, JNK2	Ulcerative colitis (UC)
Choi et al. (2023)	iNOS, NO, COX2, IL-6, IL-1β, TNF-α, p-p65, p-ERK, p-p38, p-JNK	Sepsis
Cao et al. (2019b)	CASP3, CASP6, Apaf1	Colon cancer
He et al. (2022)	CASP3, CASP12, NF-κB	Precancerous lesions
Liu et al. (2022b), Li et al. (2024b)	HMOX1, CDK1, PDK1, β-catenin	Liver cancer
Xie et al. (2024)	NO, IL-6, TNF-α, AChE, ChAT, SOD, GSH-Px	Alzheimer’s disease (AD)
Zeng et al. (2023)	CD86, HBsAg, HBcAg, iNOS	Chronic hepatitis B
Wang et al. (2022d)	TNF-α, IL-1β, IL-6, TLR2, TLR4, NF-κB-p65, p-p38 MAPK, p38 MAPK	Rheumatoid arthritis
Kim et al. (2022b)	PI3K, p-PI3K, AKT, p-AKT, p-mTORC1, mTORC1, p-p70S6K, p70S6K,p-4EBP1, 4EBP1, p-FOXO3A, FOXO3A, MuRF1, Atrogin-1, SIRT1, PGC-1a, NRF1, NRF2, TFAM	Muscle atrophy
Han and Choung (2022)	p-mTORC1, p-AKT, p-4EBP1, p-S6K1, p-FOXO3A, MuRF1, Atrogin-1, SREBP-1C, DGAT2, SCD1, CPT1, UCP3, ACOX1	Muscle atrophy
Chen and Wu (2021)	AT1R, Aldosterone, SP1, TEF, AngII, Renin, ANP, Relaxin	Water and electrolytes homeostasis
Meng et al. (2021), Wang et al. (2024b)	LOC105243318, FAM132A, RORC, 1200016E24RIK, LC3, p62, GFAP	Anti-aging
Zhang et al. (2021)	AR, PI3K, AKT, p-AKT, PTEN, FOXO1, p-FOXO1, Rb, p-Rb, E2F1, Cyclin D1, CDK4, CDK6	Prostate cancer
Li et al. (2021)	SOD, CAT, MDA, GSH, ALT, AST, CD45, a-SMA, PPAR-γ, Collagen-I	Liver injury
Seo et al. (2019)	IL-4, IL-5, IL-6, IL-13, Eotaxin 3, IgE, CD4, CD25, GATA3, IFN-γ, SOD, FOXP3, IL-10	Asthma
Zou et al. (2019)	IL-6, TGF-β, TNF-a, SIgA	Immunomodulatory
Das et al. (2019)	TXA2, GPx, SOD, p-PI3K, p-Lyn, p-PLCγ, p-ERK1/2, CD41, CD42, vWF	Hypoxia induced procoagulant state

**FIGURE 2:**
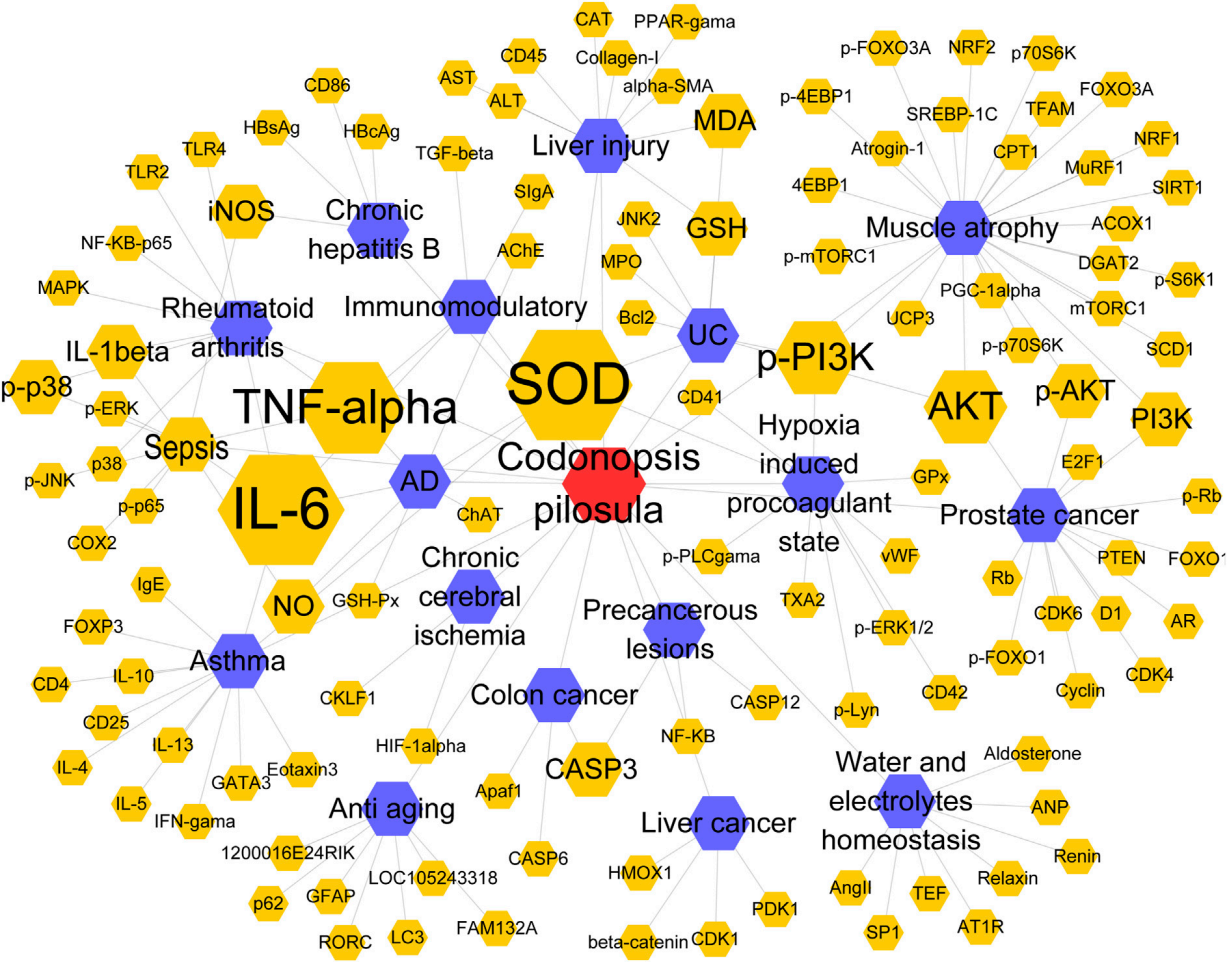
Targets of CP alone application in disease. Red represents CP. Blue represents disease. Orange represents targets.

Using cytoscape software, we constructed a network diagram of CP-disease-target gene relationships for topology analysis to uncover the key genes of CP in action diseases. The results (Figure 2) showed that the proinflammatory factors TNF-α and IL-6 were still at the highest point of the study among the genes related to CP acting diseases. In a mouse model of scopolamine-induced memory impairment, CP enhances anti-inflammatory function by inhibiting TNF-α, IL-6 and regulating intestinal flora (Xie et al., 2024).

Oxidative stress is a physiological phenomenon in which there is a state of imbalance between oxidants (e.g., free radicals, peroxides, etc*.*) and antioxidants (e.g., antioxidant enzymes, antioxidant molecules, etc.) in the environment on the inside and outside of the cell. Oxidative stress occurs when an organism’s oxidative stress capacity exceeds its antioxidant capacity. Fortunately, CP maintains the stability of the internal and external cellular environment and reduces the effects of oxidative damage on the health of the organism by protecting the structural integrity of cells, promoting the activity of antioxidant enzymes, and neutralizing free radicals (Li F. et al., 2024; Xie et al., 2024). In a mouse model of carbon tetrachloride (CCl4)-induced chronic liver injury, CP significantly attenuated liver injury and fibrosis by increasing SOD and CAT activities, decreasing MDA levels, and increasing GSH content to mitigate oxidative stress injury, as well as inhibiting intrahepatic inflammatory cell infiltration (Li et al., 2021).

The mechanism of CP action in an organism is a complex regulatory network. Proinflammatory factors and oxidative stress can affect biological processes such as cell survival, proliferation and apoptosis by activating multiple signaling pathways in the organism. The PI3K/AKT signaling pathway plays a key role in cell proliferation and metabolism (Gao W. et al., 2020). In a mouse model of muscular dystrophy, CP promotes muscle protein synthesis by activating the conversion of PI3K to phosphorylated PI3K. This activates AKT, and activated AKT is phosphorylated to p-AKT, which inhibits MuRF1 and Atrogin-1 (Kim T.-Y. et al., 2022). The MAPK signaling pathway includes signaling molecules such as ERK, JNK, p38 and others. CP may regulate biological processes such as cell proliferation by inhibiting MAPK signaling activation. In LPS-induced RAW264.7 cells Codonopsis pilosula reduced the expression of TNF-α, IL-6 and IL-1β in a dose-dependent manner. It also inhibited p-p38, p-ERK, and p-JNK in a dose-dependent manner, thus inhibiting the JNK signaling pathway to exert anti-inflammatory effects (Choi et al., 2023).

### 2.2 Studies on CP combination drugs in diseases

In traditional Chinese medicine, CP is often used in combination with other drugs (Dong et al., 2023). It plays an important role in antioxidant, anti-gastrointestinal mucosa and anti-tumor. This review summarizes the research progress of CP combination drugs in a variety of diseases in the last 5 years as shown in Supplementary Table S1.

The results showed that the combination of CP drugs mainly acted in the treatment of cardiovascular system, nervous system, tumor-related, digestive system, metabolic system and other diseases. In the drug therapy involved in CP not only relieves the symptoms of gastritis (Xu Q. et al., 2023), but also blocks the progression of gastritis to gastric cancer (Weng et al., 2023). CP has been used as an important component in the treatment of breast cancer (Xu et al., 2024), gastric cancer (Xu J. et al., 2023), and non-small cell lung cancer (Hao et al., 2015) for many years as an adjuvant therapy (Chen G. et al., 2024). In cardiovascular diseases such as arrhythmia (Wang et al., 2021), heart failure (Liu et al., 2020), and coronary heart disease (Fan et al., 2021), CP as a medicinal ingredient, can protect the heart by regulating related genes.

**FIGURE 3:**
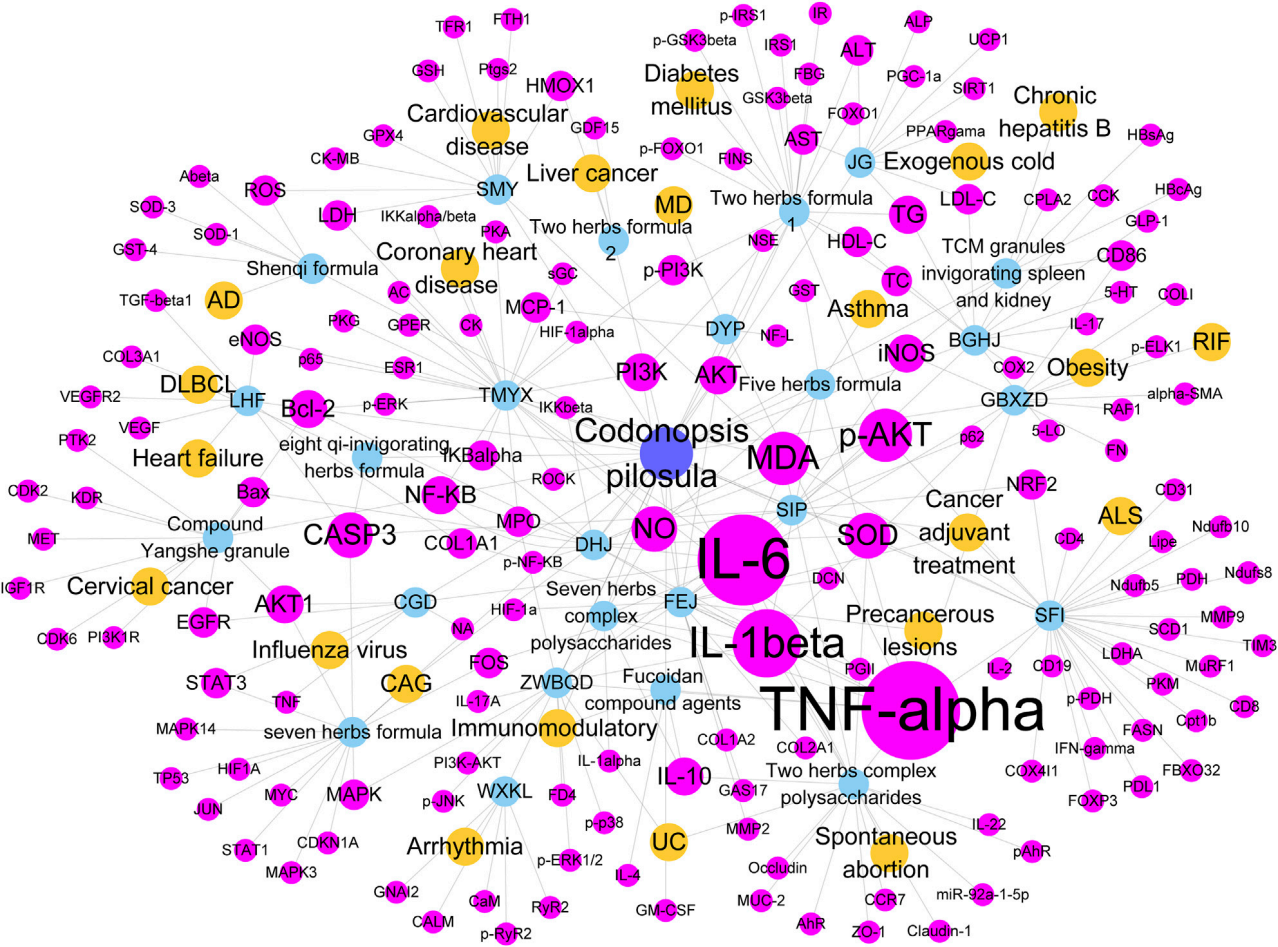
Targets of CP combination drug in disease. Blue represents CP. Orange represents disease. Azure blue represents party ginseng combined with drugs. Pink represents targets.

Cytoscape software was used to construct the network diagram of CP combination drugs-disease-target relationship and analyze the topology. The results (Figure 3) showed that among the disease-related targets, the proinflammatory factors TNF-α, IL-1β, and IL-6 were studied most frequently. These proinflammatory factors have a wide range of biological activities and help coordinate the body’s response to infections. TNF-α belongs to the TNF ligand superfamily, which is mainly secreted by macrophages and lymphocytes (Yuk et al., 2024), and promotes the production and secretion of IL-1β and IL-6. TNF-α regulates imbalances in immune regulation (Deng et al., 2020), inflammation (Xu Q. et al., 2023), cancer (Zhu et al., 2019), memory disorders (Ren et al., 2022) and other diseases. In Xu et al.’s study, Dangshen Huangjiu (DHJG) achieved the efficacy of preventing gastric mucosal injury by elevating SOD and decreasing MDA, increasing antioxidant capacity, and inhibiting the AKT/NF-κB signaling pathway to decrease the expression of inflammatory factors TNF-α, IL-1β, and IL-6 in the chronic non-atrophic gastritis model of Wistar rats (Xu Q. et al., 2023).

In addition to inflammation, excessive oxidative stress damages the gastric mucosa, leading to alterations in the endogenous antioxidant defense system (Kim et al., 2020). SOD and MDA are two of the most used oxidative stress metrics. The ROS response on the cell membrane leads to lipid peroxidation, which results in elevated levels of MDA and oxidative damage to the stomach (Cui et al., 2021). The combination of Radix Astragali polysaccharides with CP polysaccharides in a mouse model of colitis could improve colitis symptoms in mice by elevating SOD, decreasing MDA to improve antioxidant activity, and simultaneously decreasing the expression of inflammatory factors TNF-α, IL-1β, and IL-6.

CASP3 is a key member of caspases characterized by programmed cell death and is often used as a marker for cancer therapy. ZHANG et al. demonstrated that CASP3 activation triggers cellular pyroptosis, which is essential for immunomodulation by cleaving Gasdermin E (GSDME) in tumor cells (Zhang Z. et al., 2020). In a mouse model of EL4 lymphoma, cytotoxic CD8^+^ T cell-induced immunogenic cell death and diffuse immunogenesis against endogenous tumor antigens depended on CASP3-dependent apoptosis in EL4 cancer cells (Jaime-Sanchez et al., 2020). In addition, CASP3 is also thought to be a common target of anti-DLBCL apoptosis in quinonic herbs containing CP, acting on the microenvironment of DLBCL through CASP3(Huang et al., 2021).

The enzyme PI3K converts AKT into p-AKT, triggering a series of signaling cascades involved in regulating cell survival, proliferation, metabolism, apoptosis, and other biological processes. In a rat model of gastric cancer precancerous lesion (PLGC), the levels of PI3K, *p*-AKT and HIF-α were significantly upregulated, whereas the levels of PI3K, p-AKT and HIF-α were suppressed after Fufang E’jiao Jiang administration (Shi et al., 2023). The independent effects of CP(He et al., 2022) and other components of Fufang E’jiao Jiang (Chin et al., 2023; Lien et al., 2023; Omrani et al., 2024; Yang et al., 2024) in diseases have been studied. However, the strength of CP’s efficacy when used independently versus as a component of Fufang E’jiao Jiang has not been thoroughly investigated. Future studies should pay more attention to the interaction of CP in combination therapy with different drugs, the mechanism of potency enhancement, and the range of adapted cases. This will enable them to gain a deeper understanding of its clinical potential.

### 2.3 Studies on active ingredients of CP in various diseases

#### 2.3.1 Studies on CP polysaccharides (CPPs) in a variety of diseases

As a traditional medicinal plant, CP is also known as the poor man’s “ginseng” (Jolly et al., 2024). It is rich in polysaccharides, ginsenosides, alkaloids, flavonoids and other complex active ingredients (Gao et al., 2018; Bailly, 2021; Luan et al., 2021). This review summarizes the research on CP active ingredients in the last 5 years (Supplementary Table S2), and the analysis reveals that among CP active ingredients, CPPs are the most abundantly researched.

CPPs, as an important active ingredient and biomarker of CP(Luan et al., 2021; Yue et al., 2023), play important pharmacological roles in a wide range of diseases, especially metabolic diseases (Zhang Y. et al., 2020; Bai et al., 2022; Chen S. et al., 2024), digestive diseases (Meng et al., 2020; Zhou et al., 2024), hepatic diseases (Hu et al., 2022; Meng X. et al., 2023), neurological diseases (Wan et al., 2020; Hu et al., 2021) and respiratory diseases (Gong et al., 2022). In a high-fat/high-sucrose diet-induced mouse model, CPPs led to a decrease in MDA levels and an increase in the ratio of GSH to oxidized GSH, as well as an increase in SOD and CAT, which activated the antioxidant signaling pathway and ameliorated high-fat/high-sucrose diet-induced insulin resistance (Zhang Y. et al., 2020). In addition, CPPs inhibited the accumulation of lipid vesicles in the cytoplasm and the expression of markers of adipogenic differentiation (PPARγ and C/EBPα) in a concentration-dependent manner in an SD rat osteoporosis model established by bilateral ovariectomy (OVX). They also increased the expression of β-catenin, a core protein of the Wnt/β-catenin signaling pathway, which ameliorates bone loss in OVX rats *in vivo* (Liu J. et al., 2023).

**FIGURE 4:**
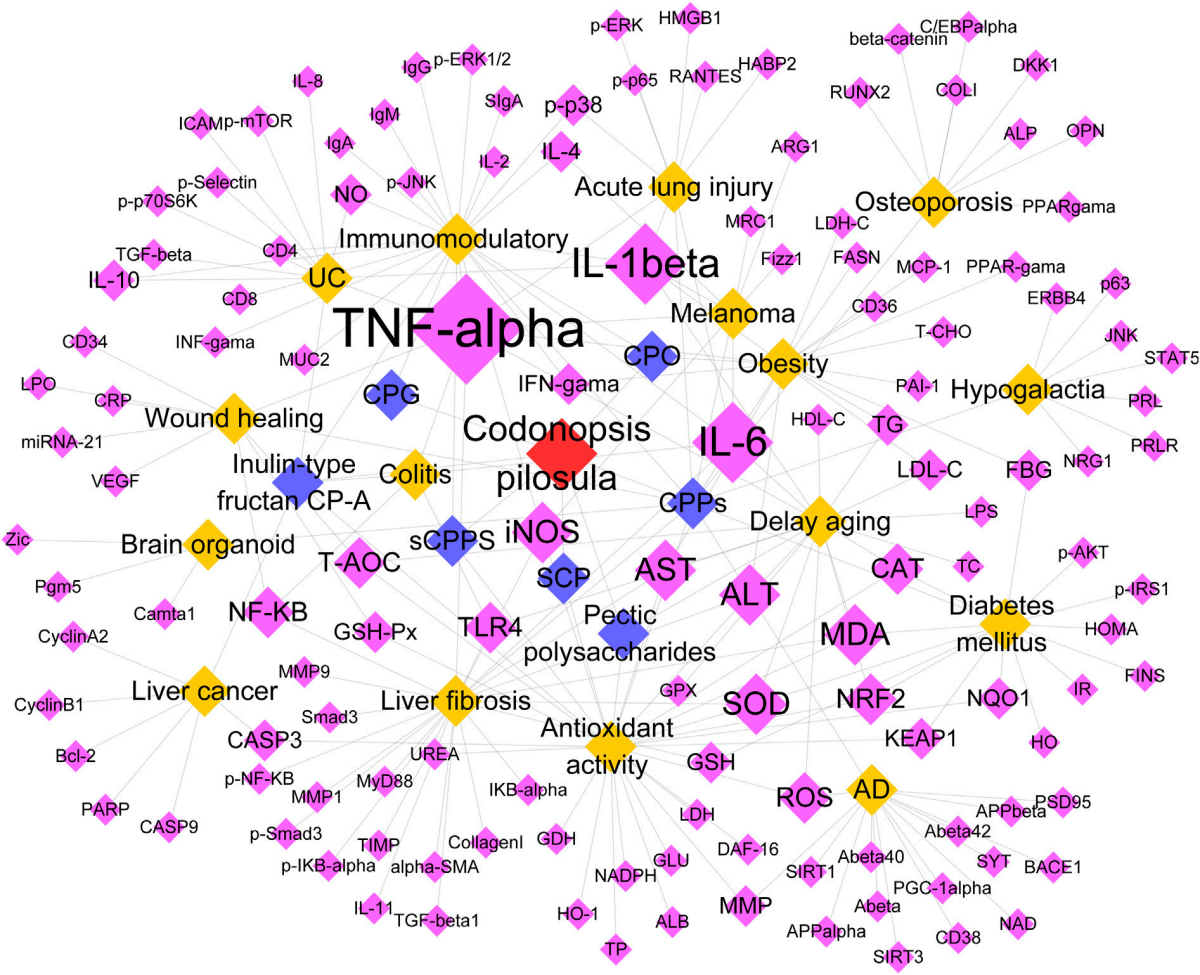
Targets of CPPs in disease. Red represents CP. Blue represents the type of CPPs. Orange represents disease. Pink represents targets.

To further analyze the key targets in CPPs, we constructed a network diagram of the relationship between CPPs-disease-target genes by cytoscape (Figure 4). The results showed that the proinflammatory factors TNF-α, IL-6, and IL-1β were still under active study. In a melanoma mouse model, CPPs inhibited IL-4 induced proliferation of M2-like tumor-associated macrophages (TAMs) and significantly increased the expression of TNF-α, IL-6, IL-1, and iNOS, which promoted the repolarization of M2-like TAMs to M1-like TAMs (Liu H. et al., 2021).

Secondly, oxidative stress is also a major pathway of action for CPPs to exert their functions. CPPs can exert anti-oxidative stress by scavenging free radicals and increasing antioxidant enzyme activity, protecting cells from oxidative damage. In the intestines of naturally aging mice, high doses of CP pectin polysaccharides significantly enhanced the expression of all antioxidant enzymes SOD, GPX, CAT and NRF2. In contrast, the levels of the inflammatory factors TNF-α, IL-1β, IL-6, and TLR4 were dose-dependently decreased (Zou et al., 2023). Therefore, CP pectin polysaccharide significantly downregulated inflammatory factors, upregulated antioxidant enzyme activities, and repaired intestinal barrier function in a dose-dependent manner (Zou et al., 2023). In addition, CP inulin-type fructans enhanced the antioxidant defense of intestinal epithelial cells by enhancing cell viability, increasing GPX, SOD, and CAT, and decreasing MDA and LDH (Zou et al., 2021).

#### 2.3.2 Studies on other active ingredients of CP in various diseases

To date, hundreds of compounds have been isolated and identified from CP(Zhang et al., 2023). In addition to CPPs, which are the main constituents and the most abundantly studied active ingredients in the last 5 years, Lobetyolin, lancemaside A, Saponins, Luteolin, Alkaloids and other constituents have also been studied by researchers. Among the diseases related to the action of these active ingredients (Table 2), Lobetyolin (He et al., 2020; Cheng et al., 2023), Luteolin (Yu et al., 2023; Liu et al., 2024), polyacetylenes (Wang M.-C. et al., 2022), Isorhamnetin (Luan et al., 2019) and molecule compound D6 (Tang X. et al., 2021) mainly act in the treatment of cancer, saponins act in digestive diseases (Liu X. F. et al., 2021; Li et al., 2023), lancemaside A (Lee et al., 2019; Shin et al., 2019) and atractylodesin III(Cao M. et al., 2019) act in cardiovascular diseases. In addition, in a mouse model of non-alcoholic fatty liver disease (NAFLD), Alkaloids attenuate lipid deposition in NAFLD by improving energy metabolism, reducing oxidative stress and endoplasmic reticulum stress, and thus act as hepatoprotective agents (Fan C. et al., 2023). Aromatic derivatives slow down carbohydrates by inhibiting alpha-glucosidase activity during digestion, management and absorption, thus helping to control blood glucose levels (Wang R.-Y. et al., 2022).

**TABLE 2:** Studies on other active components of CP in diseases.

Components of codonopsis pilosula	Related targets and genes	Functioning diseases	References
Alkaloids	MDA, SOD, GSH, p-PERK, PERK, p-IRE1α, IRE1α, ATF6, GRP78, *p*-eIF2α, eIF2α, Chop, USP14	Fatty liver	Fan et al. (2023a)
Saponins	SIgA, IgG, SOD, GSH, MDA, IL-1β, IL-6, TNF-α, IFN-γ, TLR4, NF-kB, MyD88, IκBα, COX-2, CASP3	Diarrhea	Li et al. (2023)
Saponins	SOD, MDA, IL-6, IL-10, NF-kB, TNF-α	Ulcerative colitis (UC)	Liu et al. (2021b)
lancemaside A	CASP3, CASP9, ACE2, TMPRSS2	SARS-CoV-2	Kim et al. (2022a)
lancemaside A	NOX2, MDA, eNOS, NF-kB, p38, *p*-p38, JNK, *p*-JNK, ERK, *p*-ERK, *p*-eNOS, *p*-AKT, AKT	Hypertension	Lee et al. (2019), Shin et al. (2019)
Lobetyolin	ASCT2, ROS, *p*-cMyc, *p*-GSK3β, *p*-AKT, NRF2	Gastric cancer	Cheng et al. (2023)
Lobetyolin	*p*-4EBP1, *p*-p70S6k, ASCT2, SLC1A5, GSH, ROS, CASP3, CASP9, PARP, Bax, Bcl-2, COXIV, Cytochrome C, cMyc, p-cMyc, p-AKT, p-GSK3β	Breast cancer	Chen et al. (2021)
Lobetyolin	Xanthine oxidase (XO)	Hyperuricemic	Yoon and Cho (2021)
Lobetyolin	CASP3, CASP7, PARP, GLU, GSH, ASCT2, p53, p21, Bcl-2, Bax	Colon cancer	He et al. (2020)
Lobetyolin	E-cadherin, Vimentin, MMP9	Lung cancer	Liu et al. (2022a)
Luteolin	*p*-JNK, *p*-AKT, ESR	Liver cancer	Yu et al. (2023)
Luteolin	ROS, TFR1, TRF, HO-1, NRF2, GSH, Gpx4	Cancer adjuvant treatment	Liu et al. (2024)
Codonopsis lanceolata polyacetylenes (CLP)	Ras, PI3K, *p*-AKT, Bcl-2, cyclin D1, CDK4, Bax, GSK-3 β, CASP3, CASP9	Lung Adenocarcinoma	Wang et al. (2022b)
Codonopsis pilosula molecule compound D6	EGFR, PARP, *p*-Y530, *p*-Y397, *p*-AKT, *p*-ERK1/2, HSP90, CDK4, c-Raf1, pGSK3β	Non-small cell lung cancer (NSCLC)	Tang et al. (2021b)
Codonopsis pilosula aromatic derivatives	*α*-glucosidase	Diabetes mellitus	Wang et al. (2022c)
Atractylodesin III	Bcl-2, Bax, CASP3	Myocardial infarction	Cao et al. (2019a)
Isorhamnetin	APAF1, CASP3, CASP9, Hspa1a, Hspa1b, Hspa8	Colon cancer	Luan et al. (2019)
Isorhamnetin	p-AKT, p-PI3K, p-mTOR, SOD, MDA, GSH-Px	Parkinson’s disease	Gu et al. (2020)

**FIGURE 5:**
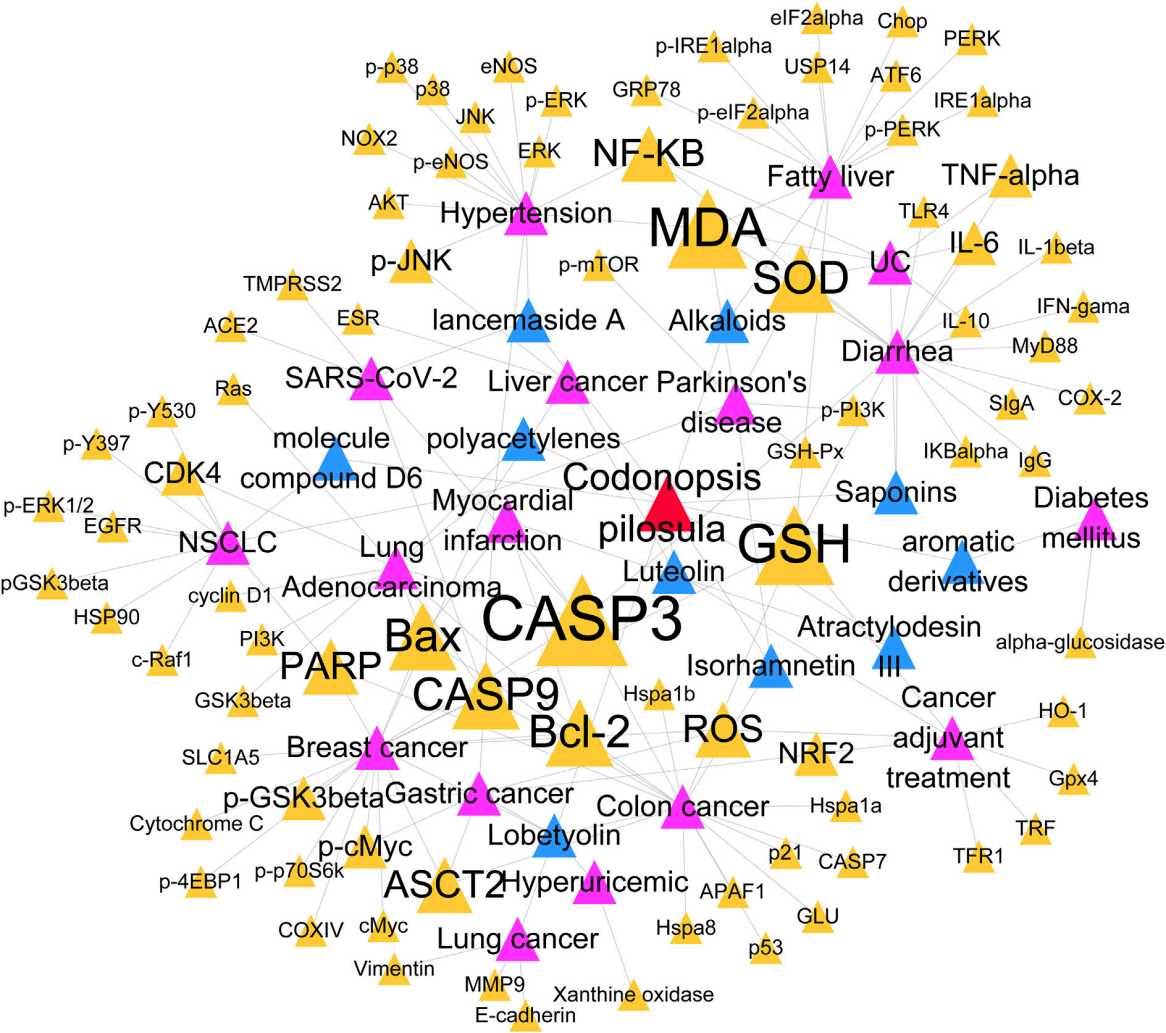
In addition to CPPs, targets of other active ingredients of CP in disease. Red represents CP. Blue represents the active ingredient of CP. Pink represents disease. Orange represents targets.

Next, a network diagram of the relationship between CP active ingredients-disease-target genes was constructed using Cytoscape software for topology analysis. The results (Figure 5) showed that apoptosis related genes CASP3, CASP9, Bax, and Bcl-2 were studied with high frequency. Apoptosis is an important mode of programmed cell death, which plays a key role in maintaining tissue homeostasis, removing damaged cells, and inhibiting tumor development. Luteolin in CP inhibits the uptake of glutamine in breast cancer cells in a dose-dependent manner, which serves as a substrate for GSH synthesis, which also leads to a decrease in GSH levels and an increase in ROS levels (Chen et al., 2021). Meanwhile Luteolin increased the cleavage of CASP3, CASP9 and PARP, promoted the release of cytochrome C from mitochondria to the cytoplasm and induced apoptosis in breast cancer cells (Chen et al., 2021). In a rat model of acute myocardial infarction, Atractylodesin III reduces apoptosis of cardiomyocytes in acute myocardial infarction by decreasing the expression of Bax and CASP3, and up-regulating the ratio of Bcl-2 and Bcl-2/Bax (Cao M. et al., 2019).

The oxidative stress pathway remains an essential mechanism of action for other active ingredients in CP. Saponins in CP can increase SOD activity and decrease MDA content in colon tissues, thus effectively scavenging intracellular superoxide radicals and reducing lipid oxidation (Liu X. F. et al., 2021). Meanwhile, it inhibited the expression of IL-6 and TNF-α in the colon, promoted the elevation of IL-10, inhibited the NF-κB signaling pathway, and moderated the symptoms of ulcerative colitis in rats (Liu X. F. et al., 2021). The NF-κB pathway is an important cell signaling pathway. It can be activated through multiple pathways (Shin et al., 2019) and also regulates the expression of multiple genes. In a hypertensive rat model, Lancemaside A decreased the expression of NF-κB, p38, p-p38, p-JNK, JNK, ERK, p-ERK, and effectively inhibited the NF-κB and MAPK signaling pathways in a dose-dependent manner to exert anti-inflammatory and antihypertensive effects (Shin et al., 2019).

### 2.4 CP medical prospects and application value

As a traditional Chinese herbal medicine, CP has a long history and wide application in traditional Chinese medicine. By systematically summarizing the literature, this review found that CP has an important role in disease research. Considering its rich pharmacological activities and diverse medicinal effects, it can be applied to medicine in a wide range of applications.

First of all, CP has the efficacy of regulating qi and blood, benefiting qi and generating fluids. In Chinese medicine theory, qi and blood are the basic sources of life energy in the human body. Qi refers to the basic substances that make up the human body. It is also the power that regulates and drives all physiological activities in the body. Blood, on the other hand, carries sustenance and nutrients for the human body’s tissues and organs. Therefore, the warmth of qi and the moistening of blood together maintain the normal physiological functions of the human body. CP is rich in polysaccharides, saponins and other active ingredients (Gao et al., 2018; Luan et al., 2021) can enhance the body’s immunity and disease resistance (Bai et al., 2020), thus playing an important role in regulating the body and enhancing immunity, etc. CP and its active ingredients can strengthen the body’s defense against external aggressions by promoting the activation of immune cells and the release of cytokines. They can also improve the body’s disease-resistant ability, which helps to maintain body health.

Secondly, CP has a protective effect on the cardiovascular system. CP can regulate the function of the cardiovascular system (Meng P. et al., 2023), including lowering blood pressure (Lee et al., 2019; Shin et al., 2019), regulating blood lipids, and controlling heart rhythm (Wang et al., 2021), thus helping to prevent and treat cardiovascular diseases. These effects may be related to the antioxidant, anti-inflammatory, and vasodilatory effects of CP and its active ingredients, which protect the health of the heart and blood vessels by improving the functional state of the cardiovascular system and mitigating the onset and progression of cardiovascular diseases.

In addition, CP has anti-tumor and anti-cancer effects (Cao Y. et al., 2019; Liu Z. et al., 2022). It can inhibit tumor cell proliferation and promote tumor cell apoptosis, as well as reduce radiotherapy side effects and improve the quality of survival of cancer patients (Zhu et al., 2019; Li W. et al., 2024). These effects may be related to the fact that CP and its active ingredients have antitumor, antioxidant and immunomodulatory effects. These effects intervene in the growth and development of tumors through a variety of pathways, enhance the body’s resistance to cancer, and improve the quality of life of patients.

Finally, CP can improve cognitive function, delay nerve aging, and protect nerve cells. It prevents and treats neurological diseases such as Alzheimer’s disease (AD) (Zhi et al., 2023; Xie et al., 2024) and Parkinson’s (Gu et al., 2020). CP and its active ingredients may improve the brain environment through antioxidant, anti-inflammatory, anti-neural cell apoptosis, etc., and promote the survival and functional recovery of nerve cells (Hu et al., 2021; Chen H. et al., 2024; Xie et al., 2024), thus protecting the nervous system’s health and slowing down the development of neurological diseases.

## 3 Discussion

Traditional Chinese medicine (TCM) is characterized by extensive resources, simple concoctions, impartial efficacy, and high economic benefits. However, due to the complexity of TCM components, the mechanism of action of a single component cannot be elucidated. This leads to limitations in TCM promotion (Cao M. et al., 2019). CP, as a nourishing, practical, and economical herb, has been widely developed as a medicine and functional food (Zeng et al., 2022).

**FIGURE 6:**
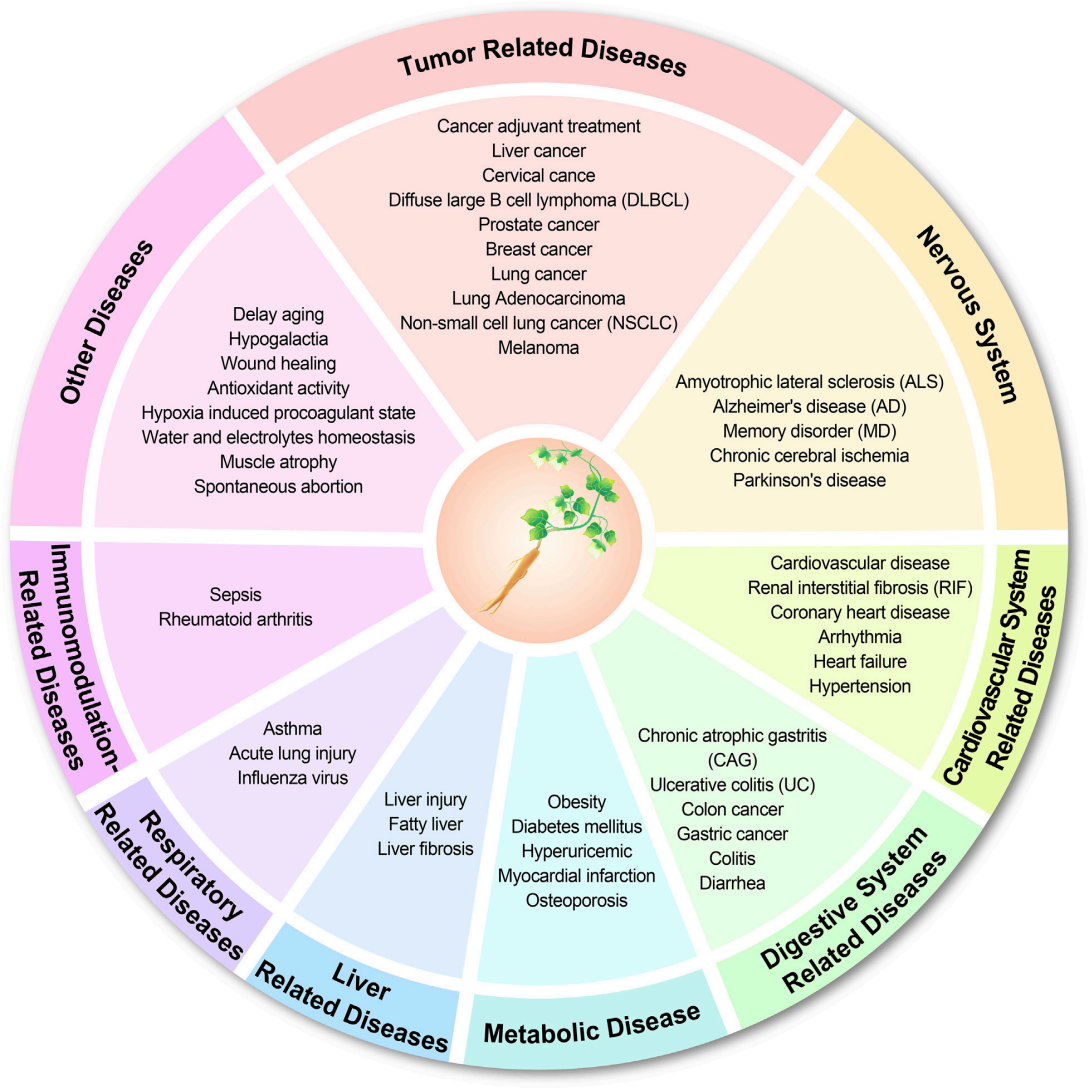
Diseases associated with the role of CP.

CP either as a stand-alone agent or in combination with other drugs, and CP active ingredients play critical roles in neurological, digestive, cardiovascular diseases, immunomodulation-related diseases and tumor-related diseases (Figure 6). ShenQi FuZheng Injection composed of CP and astragalus has been used in lung cancer, gastric cancer (Zhu et al., 2019) and chemotherapy-induced amyotrophic lateral sclerosis (ALS) (Sugimoto et al., 2021), which can effectively regulate the balance of muscle bioenergetic spectrum and effectively improve the pathological manifestations (Li W. et al., 2024). Tongmai Yangxin Pill (TMYX) containing CP(Fan et al., 2021) is effective in treating cardiovascular diseases by increasing the expression of ESR1, blocking the reduction of IκBα level and the phosphorylation of IKKα/β, IκBα, and NF-κB p65, and inhibiting the production of IL-6 and TNF-α, and exerting anti-inflammatory effects (Chen et al., 2020; 2020). When used alone as a pharmaceutical agent, CP can alleviate the symptoms of colitis (Li F. et al., 2024), hepatitis (Zeng et al., 2023), and rheumatoid arthritis (Wang Y.-J. et al., 2022) by reducing inflammation, restoring metabolic disorders, and enhancing antioxidant capacity. However, CP should be treated with more caution in prostate cancer therapy; increased activity of AR leads to increased sensitivity of prostate cancer cells to androgens, which in turn promotes PSA production, and high levels of PSA are often considered one of the indicative markers of prostate cancer. Inhibition of AR activity is currently the most effective treatment for androgen-dependent prostate cancer (Zhang Z.-B. et al., 2020). It has been shown that CP promotes prostate cancer development by enhancing AR expression (Zhang Z.-B. et al., 2020; 2021).

**FIGURE 7:**
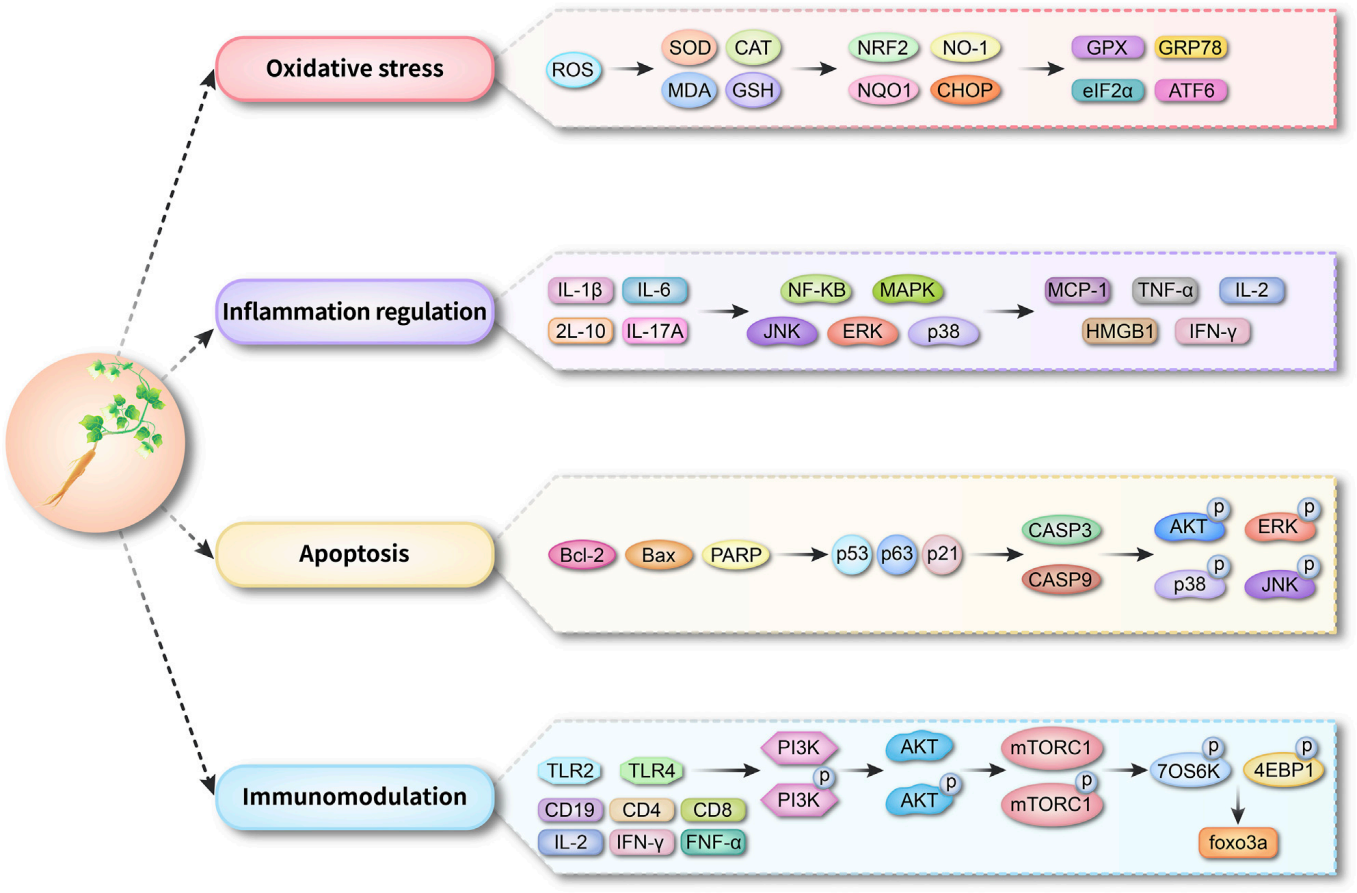
Diagram of the mechanism of CP action.

The key role of CP in disease lies in the regulation of a variety of important molecules and signaling pathways, including oxidative stress-related genes, inflammation regulation-associated genes, and apoptosis-related genes (Figure 7). These molecules influence cellular oxidative stress response, regulate inflammation level, and apoptosis process by regulating antioxidant capacity and signaling pathways such as NF-κB, PI3K/AKT, MAPK, etc., thus exerting the role of CP in diseases. CP-containing bawei guben huashi jiangzhi decoction had significant therapeutic effects on spleen-deficient obese rats by regulating MAPK and PI3K/AKT pathways through genes such as IL-6, AKT1, EGFR, ESR1, and VEGFA (Yi et al., 2024). In addition, CP alleviated colitis symptoms by blocking the activation of PI3K/AKT pathway in TNBS induced colitis in rats through the protein levels of AKT, BCL2, PI3K, and JNK2(Li F. et al., 2024). CPPs, as an important constituent of CP(Luan et al., 2021), were shown to alleviate the symptoms of colitis in an aging mouse model by decreasing the gene expression of IL-6, IL-1β, TNF-α and TLR4 gene expression, inhibiting inflammatory responses; increasing SOD, GPX, CAT, and NRF2 gene expression, reducing oxidative damage; and enhancing MUC2, Occludin, and ZO-1 gene expression, restoring the intestinal barrier, thereby delaying aging (Zou et al., 2023). CP, either alone or in combination with other drugs, appears to be associated with oxidative stress and inflammation in a variety of diseases. It has been shown that the combined administration of Astragalus and CP total polysaccharides improved colitis symptoms in mice. It upregulated IL-22 levels through AhR activation, reestablished immune balance, and attenuated mucosal damage compared with CP alone (Tang S. et al., 2021). In addition, in the rat wound model, compared with the control group, the Codonopsis pilosula crude polysaccharide (CPNP) microcapsule group and ferulic acid group had effective wound healing functions (Wang C. et al., 2022). And the expression levels of VEGF and miRNA21 were upregulated in the CPNP microcapsule group relative to the ferulic acid group. Therefore, CPNP microcapsules can exert antibacterial, anti-inflammatory and skin wound repair effects by controlled release of CPNP into the wound (Wang C. et al., 2022). However, despite some progress, CP alone and in combination still has many unanswered questions and problems to be solved.

In addition to regulating important molecules, CP can also regulate the gastrointestinal microecological balance in the organism. This is done by influencing the composition and metabolic activity of the intestinal flora and thereby regulating intestinal microecological balance. Studies have shown that Dangshen Yuanzhi Powder improves the learning and memory ability of memory-disordered (MD) animals by reducing the relative ratio of Firmicutes/Bacteroidetes and restoring intestinal flora disorder (Ren et al., 2022). Meanwhile, the regulation of blood biochemical indices by Dangshen Yuanzhi Powder in MD animals was significantly correlated with the regulation of intestinal flora (Ren et al., 2022). CP intervention reversed the abnormal levels of L-asparagine, L-glutamate, L-glutamine, serotonin hydrochloride, succinate, and acetic acid in hippocampal tissues of senescent mice (Wang X. et al., 2024), and regulated the levels of D-glutamine and D-glutamate metabolism, nitrogen metabolism, arginine biosynthesis, alanine, aspartate and glutamate metabolism, and pathways related to aminoacyl-tRNA biosynthesis, thereby slowing down the aging of the mouse brain (Wang X. et al., 2024). CPPs ameliorated splenic deficiencies in mice through significant enrichment of the probiotic bacterium *Lactobacillus* (Cao et al., 2022).

Although this review provides an in-depth discussion of the role and mechanisms of CP and synthesizes the results of a large number of studies, there are still some limitations. CP comes from a wide range of sources, and different varieties cultivated in various regions have different qualities and therapeutic effects (Zou et al., 2020; Liu X. et al., 2023). It was noted that CP produced in Daozhen County, Guizhou Province may be associated with neuroprotection, cardiovascular system improvement, tumor treatment and diabetes treatment. In contrast, CP produced in Weining County may be associated with neuroprotection and cardiovascular system improvement (Zhang et al., 2023). In addition, climatic conditions, altitude, topography, growth environment and growth years may affect CP growth and quality (Fan L. et al., 2023; Wang et al., 2023). Meanwhile, different extraction conditions and methods may also significantly affect the purity and proportion of CP active ingredients (Luan et al., 2021). For example, the anti-influenza virus effects of Chai Hu Gui Zhi Tang (CGD) extracts from different extraction methods may vary (Zhao et al., 2024). In addition, different structural modifications can affect CP’s impact on disease. It has been shown that selenated CPPs are more effective than CPPS in synergizing with PHA or LPS to promote lymphocyte proliferation and increase the ratio of CD4^+^ to CD8^+^ T cells. It also increased the serum levels of IgG, IgM, IFN-γ, IL-2 and IL-4 in mice, thus enhancing immunomodulatory activity (Gao Z. et al., 2020). Finally, although some comparative studies on CP-related medicinal plants are mentioned in the literature, the limited amount of relevant literature does not allow a comprehensive assessment of CP’s unique contribution to therapy. Therefore, despite the significant progress made in recent years on CP, there are still many opportunities and challenges in CP extraction methods, structural modifications, chemical structure, bioactivity and molecular mechanisms in other diseases and the unique contribution of CP in diseases, and we look forward to more in-depth studies in the future to fill these gaps.

This review summarizes the studies of CP in a variety of diseases. It finds that CP exhibits significant pharmacological effects on regulating immune function, protecting the cardiovascular system, antitumor and anticancer, and improving nervous system function. Its rich active ingredients such as polysaccharides, saponins and flavonoids provide the scientific basis for its wide application in the treatment of various diseases. Therefore, Codonopsis pilosula has become more prominent in traditional Chinese medicine and modern medicine. Its importance in enhancing human health and improving quality of life cannot be overstated.

## Abbreviations

AKT, Protein kinase B; CAT, Catalase; CASP3, caspase3; GSH, Glutathione; IL-1β, Interleukin-1β; IL-6, Interleukin-6; JNK, c-Jun N-terminal kinase; MDA, Malondialdehyde; NF-κB, Nuclear Factor-kappa B; PI3K, phosphatidylinositol-3-kinase; p-AKT, Phosphorylated AKT; P38, p38 Mitogen-Activated Protein Kinase; ROS, Reactive oxygen species; SOD, Superoxide; TNF-α, Tumor necrosis factor-α.
