# Supplementary material for: A systematic review of the mechanism of action and potential medicinal value of codonopsis pilosula in diseases
Source: Front Pharmacol. 2024 May 13;15:1415147. doi: 10.3389/fphar.2024.1415147 (PMC11128667; doi:10.3389/fphar.2024.1415147)
Supplement: Supplementary file 1 [file Table1.DOCX]

**Supplementary Table S1.** **Studies on CP combination drugs in diseases**

| **Name** | **The herbal  composition** | **Related Targets and Genes** | **Functioning  Diseases** | **Reference PMID No.** |
| --- | --- | --- | --- | --- |
| ShenQi FuZheng  Injection  (SFI) | Codonopsis pilosula, Astragalus | p-AKT1/2/3/, MMP9, CD31, IL-2, IFN-γ, TNF-α, CD19, CD4, CD8, PDL1, TIM3, FOXP3, MuRF1, FBXO32, FASN, SCD1, Lipe, Cpt1b, PKM, LDHA, PDH, p-PDH, Ndufb5, Ndufb10, Ndufs8, COX4I1, MDA, SOD, NRF2 | Cancer adjuvant treatment, Amyotrophic lateral sclerosis（ALS） | 30841452,  38237510,  34737697 |
| seven herbs  formula | Paeonia lactiflora, Atractylodes macrocephala, Pinellia ternata, Citrus reticulata, Codonopsis pilosula, Salvia miltiorrhiza , Coptis chinensis | CDKN1A, Casp3, STAT1, TP53, JUN, MAPK, STAT3, MAPK3, MYC, HIF1A, FOS, MAPK14, AKT1 | Chronic atrophic gastritis （CAG） | 38170849 |
| Dangshen Huangjiu  (DHJ) | Codonopsis pilosula, Huangjiu | p-AKT, p-NF-κB, NF-κB, Bcl-2, Bax, AKT, IKKβ, IκBα, NF-κB, SOD, MDA, IL-1β, IL-6, TNF-α | Chronic atrophic gastritis （CAG） | 37491882 |
| Chaihu Guizhi decoction  (CGD) | Bupleuri Radix, Cinnamomi Ramulus, Scutellariae Radix, Codonopsis pilosula, Glycyrrhizae Radix Et Rhizoma Praeparata, Pinellia ternata, Zingiberis Rhizoma Recens, Paeoniae Alba, Jujubae Fructus | NA, IL-6, STAT3, AKT1, EGFR, TNF | Influenza virus | 37838293 |
| Sheng-Mai-Yin  (SMY) | Codonopsis pilosula, Ophiopogon  japonicus, Schisandra chinensis | HMOX1, MDA, LDH, CK-MB, FTH1, TFR1, GPX4, ROS, GSH, Ptgs2 | Cardiovascular  disease | 37770231 |
| Guben Xiezhuo Decoction  (GBXZD) | Astragalus, Codonopsis pilosula, Centella asiatica, Salvia miltiorrhiza , Cuscuta chinensis, Rheum palmatum | COLI, FN, α-SMA, IL-1β, IL-6, TNF-α, CD86, iNOS, RAF1, p-ELK1 | Renal interstitial fibrosis  (RIF) | 37716490 |
| Bawei Guben Huashi  Jiangzhi Decoction  (BGHJ) | Codonopsis pilosula, Atractylodes macrocephala, Cassiae Semen, Lysimachiae Herba, Edgeworthiae Gardner Flos, Oryzae Semen cum Monasco, Nelumbinis Folium,  Alismatis Rhizoma | TC, TG, HDL-C, LDL-C, GLP-1, CCK, 5-HT, IL-6, IL-17, 5-LO, COX2, CPLA2 | Obesity | 38296174 |
| Shenqi formula | Codonopsis pilosula, Lycium barbarum | Aβ, GST-4, SOD-1, SOD-3, ROS | Alzheimer's disease（AD） | 37290734 |
| Two herbs complex  polysaccharides | Codonopsis pilosulapolysaccharides, Astragalus polysaccharides | SOD, MDA, IL-1β, TNF-α, IL-6, IL-22, IL-10, ZO-1, Claudin-1, Occludin, MUC-2, AhR, pAhR, miR-92a-1-5p, CCR7 | Ulcerative colitis (UC), Spontaneous abortion | 32822821,  35691200 |
| Zhen-Wu-Bu-Qi Decoction  (ZWBQD) | Poria, Paeoniae Alba, Atractylodes macrocephala, Zingiberis Recens, Codonopsis pilosula, Coptis chinensis | TNF-α, IL-1β, IL-6, IL-17A, MPO, FD4, p-AKT, p-ERK1/2, p-JNK, p-p38, MAPK, PI3K-AKT | Ulcerative colitis (UC) | 35240530 |
| Seven herbs complex  polysaccharides | Lentinula edodes, Ganodorma lucidum, Tremella fuciformis, Chrysanthemum,  Lycium barbarum, Codonopsis pilosula, Poria | NO, IL-1α, IL-1β, IL-6, IL-10, TNF-α | Immunomodulatory | 33002533 |
| Two herbs  formula 1 | Codonopsis pilosula, Polygonati Rhizoma | FBG, TC, TG, ALT, AST, FINS, IR, HDL-C, IRS1, p-IRS1, p-GSK3β, GSK3β, p-PI3K, PI3K, AKT, p-AKT, FOXO1, p-FOXO1 | Diabetes mellitus | 36589810 |
| Fufang E'jiao Jiang  (FEJ) | Asini Corii Colla, Panax ginseng, Rehmanniae Radix Praeparata, Crataegus pinnatifida, Codonopsis pilosula | PGII, GAS17, IL-6, IL-1β, TNF-α, PI3K, p-AKT, AKT, HIF-1a, COL1A1, COL1A2, COL2A1, DCN, MMP2, FOS | Precancerous lesions,  Cancer adjuvant treatment | 36563889,  30978457 |
| Compound Yangshe  granule | Hedyotis Diffusae Herba, Solani Lyrati Herba, Rubiae Radix et Rhizoma, Echinopsis Radix, Angelicae Sinensis Radix, Codonopsis pilosula, Atractylodes macrocephala | PI3K1R, AKT1, PTK2, KDR, CDK2, CDK6, IGF1R, EGFR, MET, Bax, Bcl-2 | Cervical cancer | 36220511 |
| Five herbs  formula | Acanthopanax sessiliflorum, Codonopsis pilosula , Dendropanax morbiferus, Allium hookeri , Raphanus sativus | GST, NO, IL-1β, IL-6, TNF-α | Asthma | 35804727 |
| Dangshen  Yuanzhi Powder  (DYP) | Codonopsis pilosula, Polygalae Radix , Poria, Coptis chinensis, Acori Tatarinowii Rhizoma | MCP-1, NF-L, NSE, TNF-α | Memory disorder (MD) | 35640741 |
| eight qi-invigorating  herbs formula | Panax ginseng, Panacis Quinquefolii Radix, Codonopsis pilosula, Pseudostellariae Radix, Astragalus, Glycyrrhizae Radix Et Rhizoma, Atractylodes macrocephala, Dioscoreae Rhizoma | CASP3 | Diffuse large B cell lymphoma (DLBCL) | 34955857 |
| Spleen-invigorating pills (SIP) | Codonopsis pilosula, Atractylodes macrocephala, Tangerine peel, Fructus aurantii immaturus, Crataegus pinnatifida, Colored malt | CASP3, iNOS, NO, NRF2, p62 | Cancer adjuvant treatment | 34245831 |
| Tongmai Yangxin Pill (TMYX) | Codonopsis pilosula, Rehmannia glutinosa, Spatholobus , Ophiopogon japonicus, Glycyrrhizae Radix Et Rhizoma , Polygonum multiflorum, Equus asinus, Schisandra chinensis, Chinemys reevesii , Jujubae Fructus, Cinnamomi Ramulus | ESR1, IKKα/β, IκBα, NF-κB, p65, MCP-1, TNF-α, IL-6, CK, SOD, NO, MDA, PI3K, p-PI3K, Bcl-2, GPER, p-ERK , HIF-1α, MPO, LDH, AC, PKA, eNOS, sGC, PKG, ROCK | Coronary heart disease | 33838287,  32619593,  36896463,  33058924 |
| Wenxin Keli decoction  (WXKL) | Codonopsis pilosula, Polygonati Rhizoma, Notoginseng Radix Et Rhizoma, Ambrum, Nardostachyos Radix Et Rhizoma | p-RyR2, RyR2, GNAI2, CALM, CaM | Arrhythmia | 34033900 |
| TCM granules invigorating  spleen and kidney | Codonopsis pilosula, Atractylodes macrocephala, Poria, Lycium barbarum, Cinnamomi Ramulus, Scutellariae Radix, Hedysarum Multijugum Maxim, Euryles Semen, Rehmannias Radix Praeparata, Cuscutae Semen, Pseudostellariae Radix, Agrimonia Eupatoria, salt‐ processed Psoralea corylifolia, Artemisiae Scopariae Herba, Coicis Semen | CD86, HBsAg, HBcAg, iNOS | Chronic hepatitis B | 37522253 |
| Luhong Formula  (LHF) | Cervus nippon Temminck, Carthamus tinctorius, Astragalus, Codonopsis pilosula, Cinnamomum cassia Presl, Lepidium apetalum Willd | eNOS, TGF-β1, CASP3, VEGF, VEGFR2, COL1A1, COL3A1 | Heart failure | 32565857 |
| Jiang Gui Fang (JG) | Zingiberis Recens, Cinnamomi Ramulus, Glycyrrhizae Radix Et Rhizoma , Pueraria, Spatholobus, Acanthopanacis cortex, Evodiae fructus, Codonopsis pilosula | TG, LDL-C, ALP, ALT, AST, UCP1, PGC-1a, PPARgama, SIRT1 | Exogenous cold | 31586693 |
| Two herbs  formula 2 | Codonopsis pilosula, Astragalus | GDF15, HMOX1 | Liver cancer | 30936938 |
| Fucoidan compound  agents | Codonopsis pilosula, Kjellmaniella crassifolia, Undaria pinnatifida, Astragalus polysaccharide | GM-CSF, TNF-α, IL-4, IL-10 | Immunomodula-tory | 30593813 |
