# Supplementary material for: A systematic review of the mechanism of action and potential medicinal value of codonopsis pilosula in diseases
Source: Front Pharmacol. 2024 May 13;15:1415147. doi: 10.3389/fphar.2024.1415147 (PMC11128667; doi:10.3389/fphar.2024.1415147)
Supplement: Supplementary file 2 [file Table2.DOCX]

**Supplementary Table S2.** **Studies on CPPs in diseases**

| **Components of Codonopsis  pilosula** | **Related Targets and Genes** | **Functioning  Diseases** | **Reference**  **PMID No.** |
| --- | --- | --- | --- |
| Codonopsis pilosula  polysaccharides(CPPs) | FBG, FINS, MDA, GSH, SOD, CAT, NRF2, HOMA, IR, p-AKT, p-IRS1, HO, NQO1, KEAP1 | Diabetes mellitus | 31669275 |
| Codonopsis pilosula  polysaccharides(CPPs) | SOD, CAT, T-AOC, MDA, ROS, DAF-16 | Antioxidant activity | 36091763 |
| Codonopsis pilosula  polysaccharides(CPPs) | IL-2, IL-4, IFN- γ, IL-6, TNF-α, IL-10, NO, IL-1β | Immunomodulatory | 36080221,  30797152,  30831168 |
| Codonopsis pilosula  polysaccharides(CPPs) | FBG, TG, T-CHO, LDL-C, LDH-C, ALT, AST | Obesity | 36014584 |
| Codonopsis pilosula  polysaccharides(CPPs) | TNF-α, IL-1β, IL-6, RANTES, HABP2, HMGB1, p-ERK, p-p38, p-p65 | Acute lung injury | 35818994 |
| Codonopsis pilosula  polysaccharides(CPPs) | VEGF, miRNA-21, CRP, NF-κB, TNF-α, CD34, GSH-Px, T-AOC, LPO | Wound healing | 35235468,  35090145 |
| Codonopsis pilosula  polysaccharides(CPPs) | Aβ, MMP, ROS, SIRT1, SIRT3, PGC-1α, CD38, NAD, APPα, APPβ, PSD95, SYT, Aβ42, Aβ40, BACE1 | Alzheimer's disease  (AD) | 34102973,  32652518 |
| Codonopsis pilosula  polysaccharides(CPPs) | PRL, PRLR, p63, ERBB4, NRG1, JNK, STAT5 | Hypogalactia | 38181915 |
| Codonopsis pilosula  polysaccharides(CPPs) | IL-1β, iNOS, IL-6, TNF-α, IL-4, MRC1, ARG1, Fizz1, | Melanoma | 34385100 |
| Codonopsis pilosula  polysaccharides(CPPs) | β-catenin, COL I, ALP, RUNX2, OPN, PPARγ, C/EBPα, DKK1 | Osteoporosis | 37739048 |
| Codonopsis pilosula  polysaccharides(CPPs) | SOD, GSH, MDA, iNOS, TNF-α, IL-6, IL-11, MMP9, MMP1, ALT, AST, α-SMA, Collagen I, TIMP, TLR4, MyD88, NF-κB, p-NF-κB, TGF-β1, Smad3, p-Smad3, IκB-α, p-IκB-α | Liver fibrosis | 37068337 |
| Codonopsis pilosula  polysaccharides(CPPs) | Zic, Pgm5, Camta1 | Brain organoid | 38074999 |
| selenizing CPPS (sCPPS) | IgG, IgM, IFN-γ, IL-2, IL-4, CD4, CD8 | Immunomodulatory | 32446900 |
| selenizing CPPS (sCPPS) | ROS, SOD, GSH-Px, MMP, CASP3, MDA, KEAP1, NRF2, NQO1, HO-1 | Antioxidant activity | 30521910 |
| sulfated Codonopsis  polysaccharides (SCP) | SOD, GDH, CAT, MDA, ROS, iNOS, ALT, AST, TP, ALB, GLU, UREA, KEAP1, NRF2, KEAP1, NRF2 | Antioxidant activity | 34694015 |
| Codonopsis pilosula  oligosaccharides(CPO) | NO, IL-2, INF-γ, p-p38, p-ERK1/2, p-JNK | Immunomodulatory | 32227014 |
| Codonopsis pilosula  oligosaccharides(CPO) | CD36, FASN, PPAR-γ, TNF-α, IL-1β, IFN-γ, PAI-1, MCP-1 | Obesity | 35816111 |
| Codonopsis pilosula  glucofructan(CPG) | IL-2, IFN-γ, TNF-α, IgG, IgM, IgA, NO, iNOS, IL-6 | Immunomodulatory | 37958581,  36934161,  35267905 |
| inulin-type fructan CP-A | IL-8, TNF-α, IL-10, TGF-β, NO, IL-1β, ICAM, p-Selectin, p-mTOR, p-p70S6K | Ulcerative colitis  (UC) | 38193197 |
| inulin-type fructan CP-A | TLR4, NF-κB, TNF-α, IL-6 | Colitis | 32979437 |
| inulin-type fructan CP-A | SIgA, MUC2, IL-1β, TNF-α | Immunomodulatory | 35237158 |
| inulin-type fructan CP-A | CAT, SOD, MDA, GLU,LDH | Antioxidant activity | 33063324 |
| inulin-type fructan CP-A | CyclinA2, CyclinB1, CASP9, CASP3, PARP, Bcl-2 | Liver cancer | 35092739 |
| Codonopsis pilosula  pectic polysaccharides | ROS, MDA, LDH, GSH-Px, CAT, SOD, NRF2, T-AOC, NADPH, NQO1, HO-1 | Antioxidant activity | 32422266,  33857333 |
| Codonopsis pilosula  pectic polysaccharides | LPS, ALT, AST, TNF-α, IL-1β, IL-6, GPX, SOD, CAT, MDA, TLR4, NRF2, T-AOC, ROS, LDL-C, HDL-C, TC, TG | Delay aging | 37760084 |
